# Preparation and Study of the Physicochemical and Functional Properties of Nano/Micromicellar Structures Containing Chokeberry Fruit Pomace Extracts Using Egg White and Egg Yolk

**DOI:** 10.3390/ijms25158405

**Published:** 2024-08-01

**Authors:** Gohar Khachatryan, Julia Pląder, Karolina Piechowicz, Teresa Witczak, Marta Liszka-Skoczylas, Mariusz Witczak, Dorota Gałkowska, Dorota Duraczyńska, Walter Hunter, Aleksandra Waradzyn, Karen Khachatryan

**Affiliations:** 1 Department of Food Quality Analysis and Assessment, Faculty of Food Technology, University of Agriculture, Balicka Street 122, 30-149 Krakow, Poland; gohar.khachatryan@urk.edu.pl (G.K.); dorota.galkowska@urk.edu.pl (D.G.); 2 Scientific Circle of Food Technologists, Faculty of Food Technology, University of Agriculture, Balicka Street 122, 30-149 Krakow, Poland; julia.plader@student.urk.edu.pl (J.P.); karolina.piechowicz@student.urk.edu.pl (K.P.); walter.hunter@student.urk.edu.pl (W.H.); aleksandra.waradzyn@student.urk.edu.pl (A.W.); 3 Laboratory of Nanomaterials and Nanotechnology, Faculty of Food Technology, University of Agriculture, Balicka Street 122, 30-149 Krakow, Poland; teresa.witczak@urk.edu.pl; 4 Department of Engineering and Machinery for Food Industry, University of Agriculture, Balicka Street 122, 30-149 Krakow, Polandmariusz.witczak@urk.edu.pl (M.W.); 5 Jerzy Haber Institute of Catalysis and Surface Chemistry, Polish Academy of Sciences, ul. Niezapominajek 8, 30-239 Krakow, Poland; dorota.duraczynska@ikifp.edu.pl

**Keywords:** functional food, micelle, encapsulation, fruit pomace, polyphenols

## Abstract

There is currently a growing interest in health-promoting foods. The beneficial effects of food on human health are actively promoted by health professionals and nutritionists. This growing awareness is influencing the increasing range of functional foods and the pursuit of more innovative solutions. Recent research indicates that spherical nanoparticles have the potential to be used as functional biomaterials in the food industry, particularly for encapsulating hydrophobic natural phytochemicals. Techniques and systems based on micro- and nano-encapsulation are of great importance in the food and pharmaceutical industries. It is of paramount importance that encapsulation materials are safe for use in food. The aim of this study was to obtain micelles containing extracts from chokeberry fruit pomace using egg yolk powder (EYP) for emulsification (as a source of lecithin) and egg white powder (EWP) for stabilisation. The structural properties of the micelles in the resulting powders were characterised using Fourier transform infrared spectroscopy (FTIR). Scanning electron microscopy (SEM) analysis confirmed the presence of spherical micellar structures between 500 and 1000 nm in size. The water activity and water content of the obtained powders were determined, and the thermal (DSC) and antioxidant properties were investigated. The results indicated that the powder with the micellar structures had a higher stability compared to the powder obtained by simple mixing without the use of encapsulation techniques.

## 1. Introduction

As the public becomes increasingly aware of ecological issues and the popularity of the ‘zero waste’ lifestyle grows, there is a growing demand for biodegradable or reusable products. Consequently, organisations that aspire to meet societal expectations are striving to optimise the utilisation of the materials employed in the production of the final product. The demand for products with a reduced environmental impact has prompted researchers and industry to utilise raw materials that are by-products of production processes. Berry pomace is a post-production waste product of the juicing industry that contains valuable bioactive compounds [1]. A case in point is chokeberry pomace, which is a rich source of antioxidants, including polyphenols (anthocyanins, procyanidins, phenolic acids, and flavanols), and other substances with health-promoting potential [2,3,4]. Among the various sources of bioactive substances, plants represent the most promising and attractive source of functional ingredients. All parts of a plant, including the skin, roots, fruits, leaves, tubers, flowers, rhizomes, and so forth, produce bioactive substances in varying concentrations [5]. Bioactive compounds are natural antioxidants that demonstrate a multitude of health benefits and anti-disease effects in the prevention and/or treatment of urinary tract infections, cardiovascular disease, coronary heart disease, metabolic and degenerative diseases, gastric ulcers, and several forms of cancer [6,7]. Despite their numerous advantages, bioactive compounds are chemically unstable and prone to oxidative degradation. The use of pure bioactive compounds is also very limited in food products and medicines due to their rapid release, poor solubility, and low bioavailability. Encapsulation can protect bioactive compounds from environmental stress, improve their physicochemical properties, and enhance their health-promoting and anti-disease effects [8,9].

Techniques and systems based on micro- and nano-encapsulation are of significant importance within the food and pharmaceutical industries. In the encapsulation process, it is first necessary to determine which type of encapsulation system is suitable for the chosen active substance. For instance, curcumin is effectively encapsulated in lipids through emulsification [10,11]. In the selection of carriers and ingredients for encapsulation, two factors are of particular importance: the type of encapsulating materials and the encapsulation technique. It is imperative that the materials employed in the encapsulation process are safe for use in food. Stable and durable capsules can be employed in the production of functional foods with designed health-promoting properties and can also be used to enrich traditional foods with bioactive substances, such as mousses, yoghurts, kefirs, and fruit juices [12,13]. The impact of food enrichment on consumer health is significant, and therefore, it is essential to plan such initiatives with great care and in alignment with national nutrition policies. It is not always the case that any given product is an appropriate material for enrichment. First and foremost, it must be widely consumed by the entire population in sufficient quantities to compensate for the existing deficit. Moreover, the consumption of the product in question should be reasonably stable and balanced in terms of quantity. Such a product should be subjected to industrial processing in order to facilitate the implementation and control of enrichment technology. Spherical nanostructures, such as micelles, are commonly employed as carriers of bioactive compounds during food enrichment.

Micelles are aggregates of nanometre-sized particles that are formed by the self-organisation of amphiphilic polymers in aqueous solution. The hydrophobic core and hydrophilic shell of these micelles render them an optimal carrier for hydrophobic functional food ingredients. The hydrophilic shell of the micelles allows for the separation of lipophilic components from the environment, while the hydrophobic core adsorbs or binds hydrophobic components. The lipophilic core of the chain (in aqueous solution) is encapsulated and stabilised by the hydrophilic shell. It can therefore be concluded that amphiphilic di- and triblock copolymers can exceed 100 nm in size and still be considered micelles [14,15,16,17]. The unique properties of micelles, including their small particle size, capacity for solubilisation, high encapsulation efficiency, and targeted release of hydrophobic bioactive compounds, have attracted considerable attention. It is for these reasons that micelles are considered an important vehicle for the encapsulation of functional ingredients, with the aim of improving their bioavailability and stability [18,19]. To illustrate, micelles can mitigate the degradation and evaporation of volatile functional ingredients, thereby enhancing the stability of sensitive compounds [20,21].

A variety of substances can be employed to facilitate the formation of micelles, with lecithin representing a notable example. It is a natural mixture of phospholipids, which is widely known for its emulsifying properties and unique technological characteristics [22,23,24]. The highest concentrations of lecithin are found in hen’s egg yolks and specific oilseeds, including soybeans and rapeseed. The components of egg yolk lecithin include phosphatidylcholine, phosphatidylethanolamine, and lysophosphatidylcholine. The lecithin content of egg yolk is approximately three times that of soy. Lecithin has been demonstrated to delay the process of ageing, to protect the stomach and liver, to promote the utilisation of fat-soluble vitamins, and to improve cardiovascular fitness. In the food industry, lecithin is employed primarily as an emulsifier to facilitate the formation and stabilisation of emulsions. It is also utilised as an antioxidant or viscosity and fluidity regulator for chocolate paste [25,26]. Chicken eggs are a rich source of lecithin, a substance that is beneficial to human health and well-being. They are a nutritious food that is also a good source of vitamins and minerals, suitable for people of all ages. Eggs contain a number of beneficial unsaturated fats and are an excellent source of choline, selenium, protein, vitamin B12, phosphorus, and riboflavin [27]. Furthermore, they exhibit multiple functions (foaming, gelling, and emulsifying), rendering them optimal and adaptable ingredients for utilisation in the food industry and domestic cooking [28]. Fresh eggs have a relatively short shelf life due to their susceptibility to microbial growth. Consequently, egg powder, particularly egg white powder, represents a valuable alternative to fresh eggs due to its significant advantages in transportation, long shelf life, and microbiological safety [29]. Egg powder is used in the bakery, confectionery, pasta, delicatessen, meat, and pharmaceutical industries. The advantages of powdered egg, apart from its extensive range of applications, include the capacity to combine it with any raw materials and confectionery and bakery semi-finished products, the maintenance of the original functional properties of hen’s eggs, and the convenience of use and storage [30].

The objective of this study was to obtain and examine the physicochemical and functional properties of nano/micromicellar structures containing extracts from chokeberry fruit pomace. Yolk powder, which serves as a source of lecithin, was employed to obtain micelles, while egg white powder was utilised for stabilisation.

## 2. Results and Discussion

**Figure 1:**
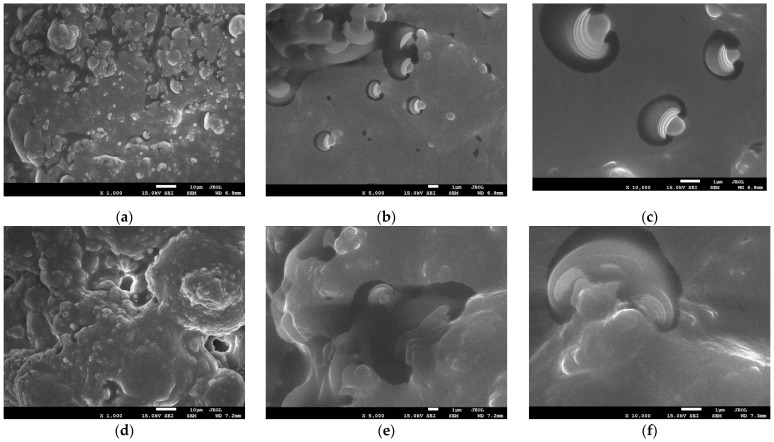
SEM images of the samples obtained: JN (**a**) ×1000 magnification, (**b**) ×5000 magnification, and (**c**) ×10,000 magnification and JE (**d**) ×1000 magnification, (**e**) ×5000 magnification, and (**f**) ×10,000 magnification.

Scanning electron microscopy (SEM) was employed to visualise the surface and morphology of the lyophilised control sample (JE) and nanostructures containing egg yolk and egg white powder (JN) samples and to characterise the resulting micellar nano/microstructures. As illustrated in Figure 1a–c, the SEM analysis revealed the presence of nano/microsphere structures in the JN sample. These structures were formed by the micellisation of the oil/extract emulsion in the lipids present in the yolk powder, which were then stabilised in the protein envelope contained in the egg white powder. The images of the predigested JE samples (Figure 1d–f) demonstrate that the pre-emulsification using ultrasound, which was performed during the preparation of the JN sample, significantly influenced the formation of regular spherical structures that are evenly distributed throughout the matrix. This is in contrast to the JE sample, where such structures are not observed.

**Figure 2:**
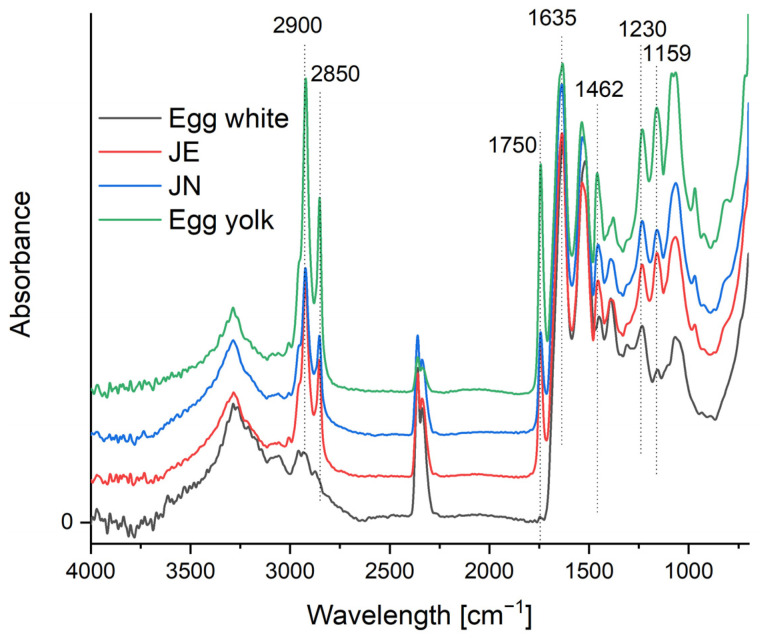
FTIR-ATR spectra of egg white, JE, JN, and egg yolk.

The types of functional groups present in the manufactured products were determined through the use of Fourier transform infrared (FTIR) spectroscopy. Infrared spectroscopy also permitted the analysis of the interactions between the groupings belonging to the individual components. The resulting spectra are presented in Figure 2.

Characteristic peaks for amide I and amide II proteins were observed in the FTIR-ATR spectra at wavelengths of 1635 and 1535 cm^−1^, respectively. These are due to C=O stretching vibrations with hydrogen bonding and C-N stretching and CNH deformation vibrations. Weaker signals at wavelengths of 1457 and 1400 cm^−1^ originate from the side chains of amino acids in peptides and proteins and are associated with asymmetric and symmetric CH_3_ deformation vibrations. The absorption band at wavelengths in the region of 1720 to 1590 cm^−1^ is attributed to the secondary structure of proteins present in chicken egg [31,32]. The presence of these bands was observed in the spectra of all samples, indicating that the protein structure was not affected by the encapsulation process.

The FT-IR spectra of chicken egg yolk show specific absorption bands around the wavelengths of 2900, 2850, 1750, 1462, 1230, 1159, and 1085 cm^−1^, which are related to the functional groups of the lipid components present in the yolk [31,33]. The presence of lipids is indicated by characteristic absorption bands in the range of 2800 to 3100 cm^−1^, caused by symmetric and asymmetric stretching vibrations of the CH_2_ and CH_3_ groups, and by an absorption band at 1750 cm^−1^, characteristic of stretching vibrations (C=O) of the ester bonds in phospholipids. The absorption peaks at lower frequencies (1230 and 1085 cm^−1^) are due to asymmetric and symmetric vibrations of the PO_2_ groups, respectively [32].

In the spectra recorded for the JN and JS samples, no significant shifts of these bands are observed, only a decrease in the absorption intensity. This indicates that the lipids present in the yolk have not been chemically modified during the emulsion preparation process. The decrease in absorption intensity for the JN samples at 2900, 2850, 1750, 1230, and 1159 cm^−1^ is probably due to the involvement of lipids in the production of the capsules.

The secondary structure of a protein is determined by the proportions of α-helices, β-sheets, β-strands, β-helices, and random turns in the peptide chains. According to the literature [34,35], changes in the secondary structures of proteins have been observed during the application of ultrasound. The transformation of an α-helix to β-sheet and the random coiling of peptide chains induced by cavitation effects in the ultrasonic field are mainly responsible for the conformational changes in protein structures.

**Figure 3:**
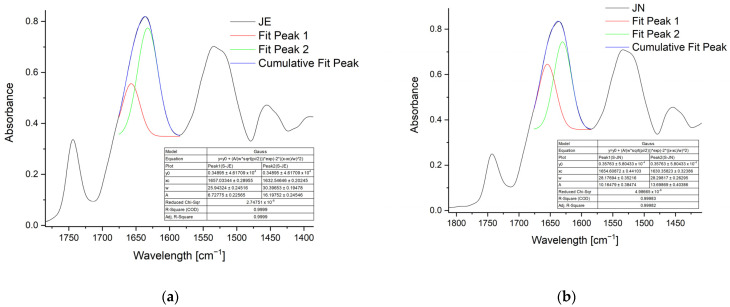
Band distribution at 1635 cm^−1^ to identify components corresponding to α-helical or β-sheet structure for JE (**a**) and JN (**b**).

In order to elucidate the changes in the secondary structure of the protein, band decomposition was performed at 1635 cm^−1^ (Figure 3).

The Figure 3 clearly shows the changes in the band intensity for the α-helical or β-sheet structure bands for amide I. The α-helical (1650 cm^−1^)-to-β-sheet (1625 cm^−1^) band area ratios for the JE and JN samples are 0.79 and 0.91, respectively. This indicates a partial configuration change in the secondary structure of the protein during the formation of spherical structures from α-helical to β-sheet.

The physical properties of the powders under investigation are contingent upon their composition, the interactions between the individual ingredients, and the type of structure that is obtained during the manufacturing process. The knowledge of the physical properties allows for the assessment of the quality and safety of food products containing them, as well as their sensory appeal. The key factors used to assess the shelf life and quality of foods are the water content and water activity and the relationship between these quantities. This relationship allows for the determination of how water molecules interact with the surface of the powder, which is employed to assess their functional properties and susceptibility to physical, chemical, and microbiological transformations. The egg white powder exhibited the highest water content and activity, followed by the egg yolk powder (EYP). No significant differences were observed between the water contents and activity levels of JE and JN, which exhibited the lowest levels of these properties (Table 1). The results obtained can be related to the drying method employed in the production of the materials in question [36]. Egg white and egg yolk powders as commercial product were obtained using the spray-drying technique, while sublimation drying was used in the preparation procedure of JE and JE powders. During spray drying, shrinkage occurs in the material as a result of the rapid removal of water, and, to a small extent, some of the water molecules are ‘trapped’ in the product matrix. In the case of freeze drying, as a result of moisture removal from freeze-concentrated glassy solids, even the water molecules bound by hydrogen bonds to the solute molecules are removed [37]. The powder formed in this way is characterised by a greater development of the surface area on which more hydrogen bonding sites will be available compared to the powder obtained by spray drying. Therefore, the freeze-dried powder will be characterised by greater hygroscopicity, which will require appropriate storage and transport conditions. Analysing the relationship between water content and water availability expressed by water activity (aw), it can be concluded that the strongest interaction of water molecules occurs for egg white powder [17], followed by egg yolk powder at 7, while the weakest interaction of water molecules was found for extract-enriched powders, respectively; for JE, it was 4 and for JN, 5. The weak water–powder interaction of JE and JN with respect to the starting scales is probably related to the presence of the hydrophobic component, which was the oil used in the preparation of the enriched egg powders. However, it should be emphasised that the activities of all the powders tested were below 0.6, which guarantees their storage stability as a result of reduced microbial growth [38].

The sensory appeal of food raw materials, food products, and the foods prepared from them represents a fundamental criterion for consumer acceptance of product choice. The initial quality parameter that consumers assess is the colour of a food’s surface. It determines his/her expectations regarding the taste and smell of food. It can be employed as a means of assessing the safety and attractiveness of food. Furthermore, it serves as a means of accepting or rejecting a specific product [39]. Two primary factors influence the colour of a food product: the colour of the raw materials used and the impact of the parameters of the technological processes applied to the raw materials on the stability of the colouring agents. The numerical data on the colour (parameters L*, a*, b*, h*, and C*) of the raw materials used (EWP and EYP) and the products produced are presented in Table 1.

The highest values of the L* parameter were recorded for the hen’s egg ingredients, indicating a higher brightness in relation to the brightness of the products obtained with them and with the dark fruit-pulp extract. The egg yolk powder exhibits a darker hue (lower L* value), a markedly higher red intensity (a* > 0), and a relatively higher yellow intensity (a* > 0) in comparison to the egg white powder. The egg yolk powder exhibits the highest colour intensity (parameter C*) and the lowest colour shade (parameter h*). The values of these parameters are consistent but not identical with those reported by Lim Jing Wei et al. [40], who studied the colour of egg yolk powder from eggs of different species of birds. The colour of the yolk in laying hens is primarily determined by the content and profile of pigments present in their feed [41]. Carotenoids, which include carotenes and xanthophylls, are yellow, orange, and red fat-soluble pigments [42]. Xanthophylls, including lutein and zeaxanthin, exert the greatest influence on yolk colour. Beta-carotene, which represents the carotenes, is present only in small quantities. The proportion of dietary carotenoids that are absorbed and deposited in the egg yolk determines the actual colour of the yolk, which varies from light yellow to dark orange [42]. Furthermore, the colour parameters of egg yolk powder are influenced by the elevated temperatures employed during its production via the commercial spray-drying method. Rannou et al. [43] observed that elevated temperatures can result in a reduction in brightness due to the Maillard reaction. The application of heat to pigment-rich foods can result in a reduction of carotenoid content by up to 50% due to the oxidation and degradation of these compounds by free radicals, which occur rapidly during the drying process [44].

Egg white, also known as albumin, is primarily composed of ovalbumin, ovotransferrin, lysozyme, ovomucoid and globulins [45]. In contrast to the yolk, it lacks pigments that impart a specific colour. In comparison to egg yolk, egg white powder is lighter (L*), more green (a*), and less yellow (b*). The obtained values for lightness (L*), redness (a*), and yellowness (b*) for egg white powder are consistent with the results of colour analysis reported in the literature [46,47]. However, there are some discrepancies between the two sets of data. The slightly higher value of the b* parameter of the analysed egg white powder in comparison to the literature data (b* = 10.06–17.87) may be attributed to discolouration of the albumen during storage. Coutts and Wilson [48] observed that prolonged storage under suboptimal conditions results in a notable increase in the yellowness of the albumen (an increase in b*). Rannou et al. [43] observed that the low brightness value (L*) of egg white powder may be attributed to a non-enzymatic browning reaction that results in the formation of dark-coloured compounds during storage.

The colours of products made from egg powder, chokeberry pomace, and rapeseed oil are markedly different to those of egg white or yolk, and the pigments in the pomace are the primary contributors to this difference. The primary class of colouring compounds identified in chokeberry fruit are polymeric proanthocyanins. The mean concentration of these compounds varies considerably, from 1578.79 mg/100 g in chokeberry juice to 8191.58 mg/100 g in pomace [49]. Anthocyanins represent the second group of colour pigments responsible for imparting colour to pomace. The darkest and reddest raw material is fruit pomace, with a lightness value of L* = 27.67, a redness value of a* = 13.905, and a yellowness value of b* = 5.07 (data not yet published).

The colour of the JN samples is statistically different from that of the JS samples in terms of all measured and calculated colour parameters with the exception of parameter C*. The JN samples exhibited darker (lower L*) and less red (lower a*) values while simultaneously demonstrating higher blue (lower b*) values in comparison to the JE samples. With regard to the C* parameter, which is indicative of colour saturation, no statistically significant difference was observed in the samples tested. Conversely, the parameter h*, which determines the tone of the colour, i.e., its hue, exhibited a higher value for the JE sample. Based on the results of the instrumental colour analysis of the prepared samples, it can be posited that some of the pigments present in the fruit pomace were encapsulated within the nanocapsules. The total colour difference (∆E*) for the JE and JN samples, calculated from the parameters L*, a*, and b*, was found to be 1.18. According to the criteria established by the International Commission on Illumination (CIE) [50], this colour difference is deemed to be negligible and unrecognisable even by an experienced and skilled observer.

**Figure 4:**
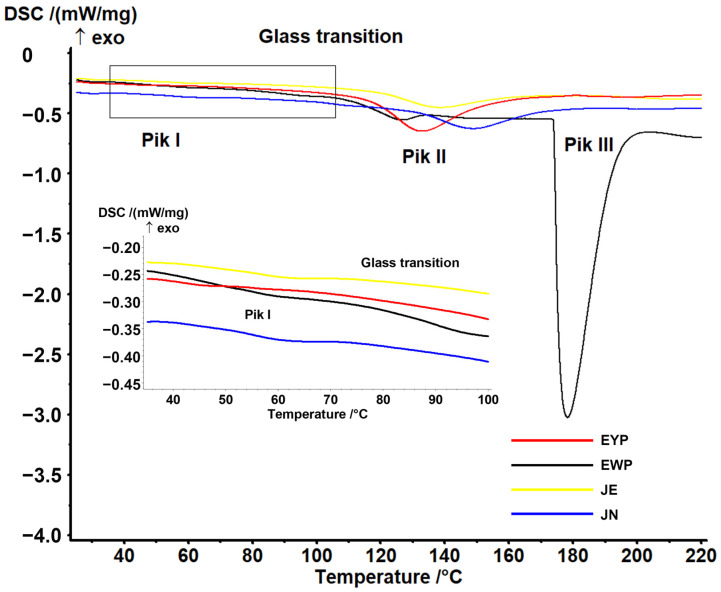
Examples of DSC curves of the samples.

Figure 4 illustrates the results of DSC analysis for the fundamental components (egg yolk and egg white) and the structures obtained through the two procedures, JE and JN. Figure 4 illustrates the curves within the temperature range of 35 to 100 °C. The data revealed the presence of minor peaks in the temperature range of 39.9 to 49.0 °C (T_on_) for the yolk, egg white, and structures. The results are presented in Table 2. No differences were identified between the JE and JN methods in this instance, and the values obtained for the transformation characteristic values were higher than those for the protein and yolk. As evidenced in prior research, the transformation associated with the denaturation of ovotransferrin falls within the following range [36,51,52]. The work of Zhao et al. [36] investigated the effect of the drying method (spray dried (SD) and freeze dried (FD)) on egg white characteristics. A peak in the range of 52.8 to 67.9 was identified, which was attributed to the denaturation of ovotransferrin. Furthermore, the authors demonstrated a correlation between the obtained values and the water content. As the water content increased, the denaturation temperature value decreased, although no value was given for the lowest values. The water contents of the structures produced (JE and JN) were found to be lower than that of egg white powder, which can be attributed to the observed differences in denaturation temperature values. A second endothermic peak was identified within the temperature range of 112 to 125.2 °C. These values correspond to the denaturation temperature values reported for ovalbumin [36,51,52]. The literature data indicate that the values range from 89.5 to 136 °C, depending on the water content. Significant differences were observed between the analysed structures (JE and JN) in terms of range and enthalpy values. This suggests that the structures produced according to the JN procedure may exhibit higher thermal stability. Nevertheless, a greater degree of variation was observed in this instance, suggesting a lack of homogeneity in the resulting preparations. No differences were observed in the characteristic temperatures, indicating that in all cases (EWP, JE, and JN), this peak is associated with the denaturation of ovalbumin.

Furthermore, a glass transition was identified for the EWP, which is consistent with the results presented in the literature [36]. Furthermore, an endothermic peak was observed in the range of 176.6 to 193.8 °C for EWP. The nature of the peak should be analysed in order to ascertain whether it is related to the melting of crystalline structures. Nevertheless, there is a paucity of literature data in this regard, and comprehensive characterisation in this instance necessitates further research. Concurrently, the disappearance of the peak in the JE and JN samples suggests an earlier disintegration of these structures as a consequence of the sample preparation procedure, which involved the addition of the extract. This may also indicate the presence of crystalline structures in the EWP.

It is well established that the intake of antioxidants through the diet plays a pivotal role in reducing oxidative damage to body cells, thereby enhancing human health. The antioxidant activity of food raw materials is contingent upon both the quantity and the composition of the bioactive compounds [53]. The majority of antioxidants derived from plants are phytochemicals, including phenols, flavonoids, and carotenoids. In contrast, the most prevalent antioxidants derived from animals are amino-derived compounds such as amino acids, peptides, and proteins [54].

Although eggs are renowned for their high nutritional value, they are not widely regarded as a valuable source of antioxidant substances. Eggs naturally contain a variety of compounds, including ovalbumin, ovotransferrin, and lysozyme in the egg white and phosphovitin, carotenoids, and free aromatic amino acids in the egg yolk. Other antioxidants, including vitamin E, carotenoids, selenium, iodine, and others, can be transferred from the feed to the egg yolk, resulting in eggs that are enriched in these compounds. It is, however, important to consider that the antioxidant bioactivity of eggs in a food product may be affected by food processing, storage, and digestion. Thermal processing methods may result in the loss of the antioxidant properties of eggs through oxidation and degradation. Conversely, the digestive process within the human body can enhance the antioxidant properties of eggs through the formation of new antioxidants, including free amino acids and peptides [55].

As anticipated, egg white and egg yolk powders exhibit minimal total polyphenol contents and demonstrate relatively low antioxidant activities with regard to both DPPH^•^ and ABTS^•+^ radical quenching and Fe(III) ion reduction (Table 3). The total polyphenol content of the protein is sevenfold lower than that of the yolk. No statistically significant difference was observed between the egg components with regard to the antioxidant activity determined using the DPPH radical and the FRAP methods. With regard to the method utilising the ABTS cation radical, the yolk exhibited a 1.6-fold higher antioxidant capacity.

A variety of plant-based foods, including fruits, vegetables, oilseeds, nuts, cereals, spices, herbs, and grains, are significant sources of antioxidants, such as phenols, flavonoids, and carotenoids. Among the numerous plant sources, the fruit of the chokeberry is renowned for its high antioxidant capacity. According to Oszmiański et al. [49], the primary class of polyphenolic compounds found in chokeberry is polymeric proanthocyanidins, accounting for 66% of the polyphenols found in the fruit. The anthocyanins present in the pomace represent the second group of phenolic compounds, accounting for approximately 25% of the total polyphenols. They are a mixture of four different cyanidin glycosides: the identified polyphenolic compounds in chokeberry pomace include 3-galactoside, 3-glucoside, 3-arabinoside, and 3-xyloside [49]. The scientific literature indicates that chokeberry pomace is considerably more rich in phenolic compounds than food products derived from the fruit [49,57,58].

Chokeberry pomace represents the second principal ingredient (after egg white and egg yolk) of the JE and JN powders subjected to analysis. Both powder samples enriched with the fruit additive exhibit a remarkably high TP content (Table 3). The TP values of approximately 300 mg/g DM for both samples are not statistically different from each other. Two of the three methods used to determine antioxidant activity (the DPPH radical and the FRAP methods) yielded higher results (statistically significant difference) for the encapsulated preparations (JN sample).

The comparable results for the total polyphenols and the slight differences in the antioxidant activities of the two types of egg powders—with encapsulated extract (JN) and with non-encapsulated extract (JE)—may suggest that the structure of the capsules constitutes a kind of barrier that inhibits the elution of polyphenols outwards. This may be related to the strong affinity of polyphenols for proteins and peptides through non-covalent interactions in the nature of electrostatic interactions, hydrophobic interactions, hydrogen bonding, and π–π stacking [59]. It can, therefore, be surmised that the protein envelope of the capsules may act as a protective agent for the antioxidant compounds present in the aronia fruit pomace extract.

## 3. Materials and Methods

The materials used were as follows: chokeberry pomace (HORTINO ZPOW Leżajsk Sp. z o.o., Leżajsk, Poland); egg yolk powder (BakePlus, Stalowa Wola, Poland); protein powder (BakePlus, Stalowa Wola, Poland); ethyl alcohol (Chempur, Piekary Śląskie, Poland), virgin rapeseed oil (Kujawski, Bunge Polska Sp. z o.o., Piekary Śląskie, Poland).

### 3.1. Extraction

Chokeberry pomace extracts were prepared using an 80% (*v*/*v*) aqueous solution of ethanol. A quantity of 5.0 g (accuracy 0.001 g) of chokeberry pomace (lyophilised, ground, and sieved) was combined with 50 mL of the extraction solution and subjected to ultrasound at a frequency of 40 Hz in an ultrasonic bath (Sonic-6D, Polsonic Palczyński Sp. J., Warsaw, Poland) for 30 min, maintaining the water temperature at 25 °C. Subsequently, the sample was subjected to centrifugation (MPW-223e, MPW Med. Instruments, Warsaw, Poland) at 3500 rpm for a period of 10 min. The supernatant was then transferred to a clean vessel. The precipitate was subsequently extracted using an additional 50 mL of the extraction solution under the aforementioned conditions. This procedure was repeated once more. The three portions of the extract were then combined and concentrated in a vacuum evaporator (RVO 200 A, INGOS s.r.o., Praha, Czech Republic) at a temperature of 40 °C. The concentrated extract was quantitatively transferred to a 50 mL volumetric flask, and the flask was made up to the mark with the extraction solution. The extract was subsequently stored at 8 °C until the nanostructures were prepared.

### 3.2. A Method for the Production of Nanostructures Utilising Egg Yolk and Protein Powder (JN)

**Figure 5:**
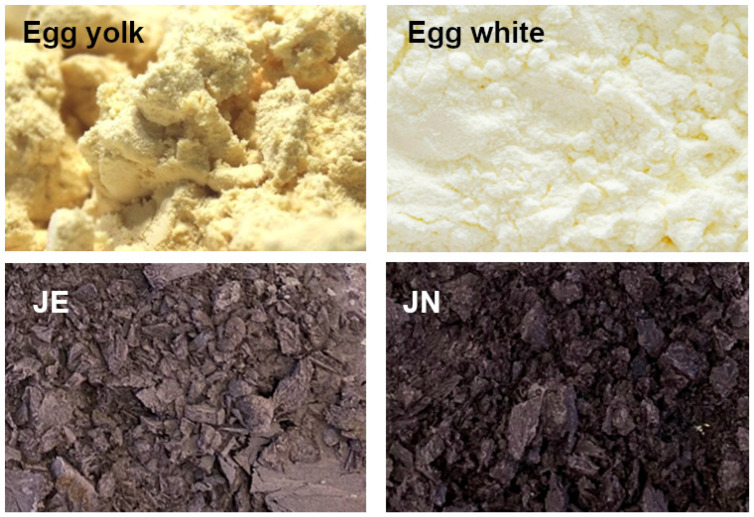
Visual comparisons of egg yolk powder, egg white, JE, and JN samples.

Firstly, 14.5 g of ethanol extract from chokeberry pomace was combined with 10 g of oil and subjected to sonification using an ultrasonic homogeniser (20 kHz, Sonopuls HD 4200, Bandelin, Berlin, Germany) until a stable emulsion was achieved. Subsequently, an aqueous suspension of egg yolk powder (8 g powder in 16 g H_2_O) was added and homogenised (20 kHz, Sonopuls HD 4200, Bandelin, Berlin, Germany). Once a homogeneous emulsion had been obtained, a suspension of protein powder (3 g powder in 27 g H_2_O) was added. The homogenisation process was continued until a stable emulsion was achieved. The resulting emulsion was then subjected to freeze drying, resulting in the JN sample (Figure 5).

### 3.3. A Method for the Control Sample (JE)

An aqueous suspension of egg yolk powder (8 g powder in 16 g H_2_O) was combined with an aqueous suspension of protein powder (3 g powder in 27 g H_2_O) using a magnetic stirrer (Heidolph RZR 2020, Heidolph Instruments GmbH & Co. KG, Schwabach, Germany). Subsequently, 10 g of oil was added, and then, 14.5 g of the ethanolic extract of aronia pomace was added to the resulting mixture in a gradual manner. The emulsion was subsequently freeze dried. The control sample (JE) was obtained (Figure 5).

### 3.4. Scanning Electron Microscopy (SEM)

The freeze-dried samples were subjected to an analysis of their nanoparticle size and morphology by means of a JEOL 7550 scanning electron microscope (Akishima, Tokyo, Japan). Prior to the measurements, the sample was coated with 20 nm chromium (Cr) using a K575X Turbo Sputter Coater (Emitech, Ltd., Kent, UK) in order to enhance the conductivity of the sample. SEM studies were conducted in high vacuum mode, and the acceleration voltage was set at 15 kV.

### 3.5. Fourier Transform Infrared (FTIR) Attenuated Total Reflection (ATR) Spectroscopy

The FTIR-ATR spectra of the freeze-dried samples were analysed at a wavelength range of 4000–700 cm^−1^ using a MATTSON 3000 FTIR spectrophotometer (Madison, WI, USA), equipped with a 30SPEC 30 Degree Reflectance accessory (MIRacle ATR, PIKE Technologies Inc., Madison, WI, USA). All measurements were conducted at a temperature of 25 ± 2 °C. The band distribution at 1635 cm^−1^ was performed using Gaussian function fitting, employing Origin 2015 software (OriginLab.com) for this purpose.

### 3.6. Determination of Dry Matter

The moisture content was determined using the thermogravimetric technique with an OHAUS MB45-45 (OHAUS Europe GmbH, Nänikon, Switzerland) drying balance. The measurement was conducted using a direct method based on the loss of mass during drying at 105 °C. A halogen heater was employed to heat the sample, facilitating the rapid completion of this process. A sample of approximately 3 g was placed on the weighing pan of a weighing machine and dried to a constant weight.

### 3.7. Determination of Water Activity

The water activity (aw) of the powders was determined using an AQUALAB 4TE (Addium Inc., Pullman, WA, USA) water activity-measuring instrument with a dew point detector using a photoelectric sensor. The tests were carried out in a thermostated chamber with a thermoelectric cell at 20 °C. The powder was placed in a vessel in an amount corresponding to half its volume. The average measurement time was five minutes, with determinations made in at least two repetitions with an accuracy of 0.003 aw.

### 3.8. Colour Determination

The colour parameters of the tested powders were determined in the CIE L*a*b* system, which has been standardised by the International Commission on Illumination (CIE) [ISO 11664-4:2008(E)/CIE S 014-4/E:2007] [60]. The measurements were performed using the reflection method on a MINOLTA CM-3500d spectrophotometer (Konica Minolta Sensing, Osaka, Japan) using illuminant D65, a measurement angle of 10°, and a 30 mm diameter membrane. The powder was placed in transparent measuring cuvette (6 cm in diameter and 4 cm in height), and the colour of the powder layer surface was measured directly at five different locations. The measurement enabled the determination of the values of the following three colour components:

The colour lightness (L*) is defined as the distance from black (L* = 0) to white (L* = 100). The a* component represents the contribution of green (a* < 0) or red (a* > 0) colour. The b* component represents the contribution of blue (b* < 0) or yellow (b* > 0) colour.

The quantitative attribute of colour is chroma (C*), which is employed to ascertain the disparity in hue in comparison to a grey colour of equivalent luminosity. A higher value of the C* parameter indicates a greater intensity of colour perception by humans. The value of the C* parameter was determined using the following formula (Equation (1)):

(1) $$C^{*}=\sqrt{a^{*2}+b^{*2}},$$

Hue (or hue angle) (h*), which is considered an attribute of colour quality, indicates the difference between a specific colour and grey of the same lightness. This parameter is defined by the following formula (Equation (2)):

(2) $$h^{*}={tan}^{-1}\left(\frac{b^{*}}{a^{*}}\right)$$

The tests were performed in quadruplicate.

### 3.9. Thermal Analysis

The thermodynamic characterisation of the analysed samples was performed using differential scanning calorimetry (DSC) with a DSC 204F1 Phoenix apparatus (Netzsch, Germany). The samples (approximately 15 mg) were hermetically sealed in aluminium vessels and then heated from 25 °C to 250 °C. The characteristic temperatures of the transformations (T_onset_, T_mid_, T_inf_, T_end_, and T_p_) and, potentially, the change in heat capacity (ΔC_p_) and the heat of fusion (ΔH) were determined using Proteus Analysis software (v. 4.8.2, Netzsch, Selb, Germany). The tests were conducted in duplicate.

### 3.10. Antioxidant Properties

#### 3.10.1. Preparation of Extracts of Test Samples for the Determination of Polyphenol Content and Antioxidant Activity

Extracts for the quantification of total polyphenols and antioxidant (antiradical) activities of the egg powders and egg powders enriched with JE and JN pomace extracts were prepared as follows: One gram of the sample was weighed to the nearest 0.001 g into a flat-bottomed ground flask and then mixed with 20 cm^3^ of 80% (*v*/*v*) methanol (Chempur, Poland). The prepared samples were boiled at 60 °C under a reflux condenser for 30 min. Subsequently, the samples were filtered through a tissue filter into volumetric flasks (50 cm^3^), which were then topped up with an 80% (*v*/*v*) methanol solution. The filtered solutions were centrifuged at 1500 rpm for 10 min (centrifuge MPW—350R, MPW Med. Instruments, Warsaw, Poland). The prepared extracts were stored at −20 °C until analysis.

#### 3.10.2. Total Polyphenol (TP) Content

The total polyphenol content of the methanol extracts of the samples was determined according to the procedure described by Singleton et al. [61]. A volume of 0.5 cm^3^ of the extract was combined with Folin–Ciocalteau reagent (0.125 cm^3^) (Chempur, Piekary Śląskie, Poland) and 25% sodium carbonate solution (0.25 cm^3^) (Eurochem, Gdansk, Poland). Following a one-hour incubation period at room temperature in the dark, the absorbance at 760 nm was determined on a Hitachi U-2900 UV-Vis spectrophotometer (Hitachi, Tokyo, Japan). The total polyphenol content was calculated using a standard curve prepared for (+)—catechin. The measurement was conducted in quadruplicate, and the result was expressed in mg/g dry weight of the test material.

#### 3.10.3. Antioxidant Activity Using DPPH and ABTS^•+^ Radical Methods and FRAP Assay

The antioxidant activities of the methanolic extracts of the tested samples were determined using the radical cation ABTS^•+^ (2,2′-azinobis-3-ethylbenzothiazoline-6-sulphonic acid) (Sigma-Aldrich, St. Louis, MO, USA) and the free radical DPPH^•^ (diphenylpicrylhydrazyl) (Sigma-Aldrich, USA) according to the procedure described by Miller et al. [62] and Brand Williams [63]. When measuring the antioxidant capacity of the test material against the DPPH radical, the absorbance at 516 nm (Hitachi U-2900 UV-Vis spectrophotometer, Hitachi, Tokyo, Japan) was measured 10 min after the addition of the radical solution (3 cm^3^ of 0.1 M DPPH^•^) to 1 cm^3^ of the extract. When the second type of radical was used, the absorbance was measured at 734 nm (Hitachi U-2900 UV-Vis spectrophotometer, Hitachi, Tokyo, Japan) 10 min after mixing 1 cm^3^ of extract and 2 cm^3^ of ABTS^•+^ radical solution. The sample solutions were diluted if necessary. Each measurement was carried out in four replicates. The antioxidant activity was expressed as µM Trolox per gram dry weight of the test material (µM Trolox/g d.m.).

The evaluation of antioxidant activity using the FRAP method (Sigma-Aldrich, USA) was based on the procedure of Benzi and Strain [64]. A 3.6 cm^3^ FRAP reagent (acetate buffer, Chempur, Poland), ferric chloride FeCl_3_ (Lach-Ner, Neratovice, Czech Republic), and TPTZ (2,4,6-tris(2-pyridyl)-1,3,5-triazine solution) (Sigma-Aldrich, Buchs, Switzerland) mixed in a volume ratio of 10:1:1 was added to 0.4 cm^3^ of methanolic extract. The sample was incubated at 37 °C for 10 min, and then, the sample was cooled and centrifuged, and its absorbance was measured at 595 nm using a Hitachi U-2900 UV-Vis spectrophotometer (Hitachi, Tokyo, Japan). The assay was repeated four times. The results were expressed as mmol Fe^2+^ per g of dry matter of the material (mmol Fe^2+^ per/g d.m.).

### 3.11. Statistical Analysis

In order to determine statistical differences between the means, the data were treated using a one-factor analysis of variance, and Duncan’s test was calculated at the 0.05 level of significance. Student’s *t*-test was used for comparisons between two samples. All calculations were performed with the statistical software package Statistica 13.0 (StatSoft Inc., Tulsa, OK, USA).

## 4. Conclusions

Electron microscopy analysis confirmed the presence of spherical micellar structures between 500 and 1500 nm in size in the obtained biocomposites. FTIR-ATR analysis confirmed that during the formation of the spherical structures (capsules), there is a partial change in the secondary configuration of the proteins from α-helical to β-sheet.

All powders tested exhibited a water activity level that ensured their microbiological stability. The differing water contents were influenced by the drying method employed and the weaker interaction of water molecules with the surfaces of JE and JN powders due to the presence of a hydrophobic component, namely oil.

The disparities in transformation temperatures exceeding 100 °C suggest the enhanced structural stability of the nanostructures/nanoencapsulates (JN) formed via the specified procedure.

The comparable results for the total polyphenol contents and the slight differences in the antioxidant activities of the two types of egg powders, JN and JE, suggest that the structure of the capsules represents a kind of barrier that inhibits the elution of polyphenols to the outside. This may be attributed to the strong affinity of polyphenols for proteins and peptides through non-covalent interactions.

The encapsulation of fruit extracts in a protein envelope appears to exert a protective effect on the antioxidant compounds present in the extract, as evidenced by the darker hue observed in the egg powders with encapsulated extract (JN).

## Figures and Tables

**Table 1:** Parameters relating to the water content, water activity, and colour of the powders.

Type of Powder	Water Content	Water Activity	Colour Parameters				
	g Water/100 g d. m.	(-)	L*	a*	b*	C*	h* (°)
EYP	2.23 ± 0.06 ^b^	0.3242 ± 0.0060 ^b^	80.65 ± 0.20 ^c^	10.75 ± 0.18 ^d^	36.57 ± 0.18 ^d^	38.12 ± 0.20 ^c^	73.62 ± 0.08 ^a^
EWP	7.10 ± 0.25 ^a^	0.3977 ± 0.0023 ^c^	91.70 ± 0.10 ^c^	−0.71 ± 0.03 ^a^	18.10 ± 0.12 ^c^	18.12 ± 0.12 ^b^	92.26 ± 0.11 ^b^
JE	1.08 ± 0.06 ^c^	0.2695 ± 0.0008 ^a^	36.26 ± 0.30 ^b^	1.92 ± 0.21 ^c^	−0.43 ± 0.08 ^b^	1.97 ± 0.19 ^a^	347.27 ± 3.63 ^d^
JN	1.24 ± 0.05 ^c^	0.2526 ± 0.0081 ^a^	35.16 ± 0.38 ^a^	1.61 ± 0.12 ^b^	−0.74 ± 0.05 ^a^	1.77 ± 0.09 ^a^	335.11 ± 3.13 ^c^
ANOVA—*p*	*p* < 0.001						

* Mean of three replicates ± standard deviation. Means signed with the same letter in certain columns are not significantly different at the 0.05 confidence level.

**Table 2:** Parameters characterising thermodynamic transformations.

	Sample	Unit	EWP	EYP	JE	JN	Test *t*—*p*
Pik I	T_on_	[°C]	41.5 ± 2.2	39.9 ± 2.4	48.4 ± 0.1	49.0 ± 1.2	0.586
	T_p_	[°C]	57.0 ± 1.3	46.6 ± 2.1	61.1 ± 0.6	61.6 ± 1.1	0.625
	T_end_	[°C]	70.8 ± 0.3	53.6 ± 4.0	73.2 ± 0.9	73.0 ± 1.1	0.898
	T_end_–T_on_	[°C]	29.3 ± 2.4	13.2 ± 0.7	24.8 ± 0.8	24.1 ± 2.3	0.726
	ΔH	[J·g^−1^]	0.776 ± 0.135	0.182 ± 0.037	0.732 ± 0.032	1.129 ± 0.170	0.083
GT	T_on_	[°C]	81.0 ± 1.0	nd	nd	nd	
	T_mid_	[°C]	88.5 ± 1.3	nd	nd	nd	
	T_inf_	[°C]	89.2 ± 0.6	nd	nd	nd	
	T_end_	[°C]	94.3 ± 1.4	nd	nd	nd	
	T_end_–T_on_	[°C]	13.3 ± 0.3	nd	nd	nd	
	ΔC_p_	[J·g^−1^·K^−1^]	0.122 ± 0.014	nd	nd	nd	
Pik II	T_on_	[°C]	112.0 ± 2.0	119.6 ± 3.1	117.6 ± 0.4	125.2 ± 9.2	0.363
	T_p_	[°C]	125.0 ± 0.2	137.7 ± 6.3	138.1 ± 0.2	142.0 ± 9.6	0.620
	T_end_	[°C]	133.5 ± 0.4	160.8 ± 8.7	164.5 ± 0.2	165.9 ± 8.6	0.834
	T_end_–T_on_	[°C]	21.4 ± 2.1	41.2 ± 6.1	46.9 ± 0.2	40.7 ± 0.6	0.005
	ΔH	[J·g^−1^]	6.7 ± 1.1	49.5 ± 7.9	24.4 ± 1.5	29.4 ± 0.1	0.045
Pik III	T_on_	[°C]	176.6 ± 6.0	nd	nd	nd	
	T_p_	[°C]	182.5 ± 6.0	nd	nd	nd	
	T_end_	[°C]	193.8 ± 5.4	nd	nd	nd	
	T_end_–T_on_	[°C]	193.8 ± 5.4	nd	nd	nd	
	ΔH	[J·g^−1^]	164.3 ± 6.5	nd	nd	nd	

Abbreviations: nd—not detected, GT—glass transition, T_on_—onset of transformation, T_p_—peak of of transformation, T_end_—endpoint of transformation, ΔH—heat of transformation, T_mid_—midpoint of glass transition, T_inf_—inflection of glass transition, ΔC_p_—change in heat capacity.

**Table 3:** Antioxidant activity.

Type of Powder	Total Polyphenol Content mg/g s.m.	Antioxidant Activity		
		DPPH^•^ µM Trolox/g s.m.	ABTS^+^ µM Trolox/g s.m.	FRAP mmol Fe^2+^/g s.m.
Egg yolk	71.26 ± 5.376 ^b^	1.99 ± 0.092 ^a^	17.48 ± 0.139 ^b^	0.88 ± 0.023 ^a^
Egg white	17.86 ± 0.405 ^a^	0.28 ± 0.009 ^a^	10.90 ± 0.122 ^a^	0.83 ± 0.018 ^a^
JE *	303.19 ± 16,56 ^c^	135.73 ± 2.583 ^b^	263,17 ± 1.752 ^c^	46.20 ± 2.420 ^b^
JN *	297.11 ± 7.794 ^c^	142.93 ± 7.283 ^c^	268.03 ± 10.675 ^c^	48.69 ± 1.282 ^c^
ANOVA—*p*	*p* < 0.001			

* Previously published results [56]. Mean of four replicates ± standard deviation. Means signed with the same letter in certain columns are not significantly different at the 0.05 confidence level.

## Data Availability

The data presented in this study are available on request from the corresponding author.
